# West Nile and Usutu virus seroprevalence in Hungary: A nationwide serosurvey among blood donors in 2019

**DOI:** 10.1371/journal.pone.0266840

**Published:** 2022-04-08

**Authors:** Anna Nagy, Nikolett Csonka, Mária Takács, Eszter Mezei, Éva Barabás

**Affiliations:** 1 National Reference Laboratory for Viral Zoonoses, Division of Microbiological Reference Laboratories, National Public Health Center, Budapest, Hungary; 2 Institute of Medical Microbiology, Semmelweis University, Budapest, Hungary; 3 Department of Communicable Diseases Epidemiology and Infection Control, National Public Health Center, Budapest, Hungary; 4 Confirmatory Laboratory, Hungarian National Blood Transfusion Service, Budapest, Hungary; Qatar University, QATAR

## Abstract

In Hungary, *West Nile virus* (WNV) has been responsible for 459 laboratory confirmed human cases between 2004 and 2019, while the first human *Usutu virus* (USUV) infection was confirmed only in 2018. A comprehensive serosurvey was conducted among blood donors to assess the WNV and USUV seroprevalence in 2019, one year after the largest European WNV epidemic. Altogether, 3005 plasma samples were collected and screened for WNV and USUV specific Immunoglobulin G (IgG) antibodies by Enzyme–Linked Immunosorbent Assay (ELISA). All reactive samples were further tested for *tick-borne encephalitis virus* IgG antibodies by ELISA. Indirect immunofluorescence test and microneutralization assay were used as confirmatory methods. Overall, the WNV seroprevalence was 4.32%, and in five blood donors USUV seropositivity was confirmed. The highest seroprevalence was measured in Central, Eastern and Southern Hungary, while the Western part of the country proved to be less affected. There was a statistically strong association between the WNV seroprevalence of 2019 and the cumulative incidence in the period of 2004 and 2019 calculated for every NUTS 3 region. The last WNV serological screening was performed in 2016 and the prevalence of anti-WNV IgG proved to be 2.19%. One year after the 2018 WNV outbreak, a significant increase in seroprevalence was observed in the Hungarian population and evidence for USUV seropositivity was also obtained. The spatial pattern of seroprevalence can support the identification of high-risk areas raising awareness of the need for increased surveillance, such as screening vector, equine, and avian populations. The communication with general practitioners and other professionals in primary health care services can support the early identification of acute human cases. Education and awareness-raising on the importance of protection against mosquito vectors amongst residents are also important parts of preventive measures.

## Introduction

*West Nile virus* (WNV) and *Usutu virus* (USUV) are phylogenetically closely related mosquito-borne members of the *genus Flavivirus*, *family Flaviviridae* [1]. Both viruses are maintained throughout nature in an enzootic cycle among different avian species as amplifying hosts and *Culex* mosquitoes as the major vectors [2]. Humans and other mammals may be infected by mosquito bites; however, they are considered incidental or dead-end hosts due to the low level of viraemia, which is not sufficient for further vector-borne transmission. The risk of WNV and/or USUV transmission via blood transfusion has also been described highlighting the requirements of blood transfusion services for preventive measures to ensure blood safety. Good manufacturing practice (GMP) regulations provide the continuous safety of blood products during the year and the screening of blood supplies could mean an additional active defense tool during summer and early autumn when competent vectors are active and abundant [3, 4]. Occupational exposure, congenital infections and transmission through breastfeeding have rarely been described [5, 6]. In most human cases, WNV infection is asymptomatic, while approximately 20–30% of infected people develop flu-like syndrome, including symptoms, such as headache, myalgia, arthralgia, and maculopapular exanthema. West Nile neuroinvasive disease (WNND) presenting as meningitis, encephalitis or acute flaccid paralysis is a potential complication of the infection, especially in elderly or immunocompromised individuals and in patients with underlying diseases [7–10]. Initially, USUV was considered to be a negligible human pathogen as only a limited number of human cases has been reported. In recent years, it has become a public health concern and clinically manifested human infections show many similarities to West Nile fever (WNF) or WNND [2, 11–14]. Various clinical signs have already been described ranging from milder symptoms to severe neurological complications, such as meningitis, encephalitis, polyneuritis, or facial paralysis [15–19]. WNV and USUV belong to the Japanese encephalitis serocomplex of the *Flavivirus genus*, sharing cross-neutralization antibodies which may complicate the laboratory differential diagnosis leading to the misinterpretation and therefore the underestimation of WNV and USUV-related disease [20, 21].

In Hungary, at least three flaviviruses are endemic: WNV, USUV and tick-borne encephalitis virus (TBEV), which clinical presentations show considerable similarities [22–25]. The laboratory diagnosis of human arbovirus infections across the entire country is performed at the National Reference Laboratory for Viral Zoonoses, National Public Health Center (NPHC), Budapest, Hungary. Diagnostic pipeline includes different serological methods and molecular tools, as well as virus isolation. The accurate laboratory diagnosis is essential due to the overlapping endemic areas and the serological cross-reactivity among flaviviruses. Therefore, specimens with the clinical suspicion of acute flavivirus infection are always parallelly investigated for TBEV, WNV and USUV immunoglobulin M, G and A (IgG, IgM, and IgA) antibodies to avoid the misinterpretation of the serological results due to the cross-reactivity among flaviviruses. Furthermore, reverse transcription real-time PCR is applied in a multiplex system to simultaneously detect WNV and USUV nucleic acid ensuring the most accurate laboratory diagnosis.

The aim of this current study was to estimate the prevalence of WNV and USUV in a representative number of blood donors from the entire territory of Hungary to assess the exposure of the population, following the 2018 WNV epidemic. Samples were tested for the presence of WNV and USUV specific IgG antibodies as serological markers for natural infection in the past.

## Materials and methods

### Study design

The population of Hungary was estimated to be 9 772 756 individuals in 2019, based on the data of the Hungarian Central Statistical Office (HCSO). Healthy blood donors between ages 18 and 65 were considered as the study population. EDTA samples of a total of 3005 voluntary whole-blood donors taking part in blood donations were enrolled in the study between: 02.04.2019 and 25.01.2020. All tests were performed on samples collected in standard donation procedure; thus, no additional serum samples were collected. Blood samples were collected from the whole territory of the country according to NUTS (Nomenclature of Territorial Units for Statistics) classification of Hungary, which designates 20 NUTS 3 regions, including 19 counties and the capital city, Budapest. Blood samples were collected across NUTS 3 regions, stratified by sex (male and female) and three age brackets (18–34; 35–50; 51–65). Sample size and distribution in each stratum was calculated according to the data of HCSO published in 2018. The study population represented 0.05% of the population in each stratum. The number of male and female blood donors was 1504 and 1501, respectively and the number of tested individuals in each age bracket was: n = 976 (18−34), n = 1130 (35−50), and n = 899 (51−65).

### Ethics statement

The study was approved by the Ethics Committee of the Hungarian National Blood Transfusion Service. The institutional Ethics Committee approved the laboratory testing of the anonymized specimens. All the applied methods were specified in a research plan. Institutional reference number of the ethical approval: *2019*.*03*.*18*. *OVSZ Etikai Bizottság*.

### Laboratory methods

Serological testing was carried out at the National Reference Laboratory for Viral Zoonoses (NPHC; Budapest, Hungary). All donor samples were tested for IgG antibodies against WNV and USUV by commercially available ELISA kits (EUROIMMUN Medizinische Labordiagnostika AG, Lübeck, Germany). The assays were performed according to the manufacturer’s instructions. The calculation of the results strictly followed the Euroimmun’s protocol. Results were evaluated semiquantitatively by calculating the ratio of the extinction value of the control or patient sample over the extinction value of the calibrator 2. Interpretation of the results met the recommendations of Euroimmun’s instructions. Plasma samples with the ratio of ≥1.1 were evaluated as positive for WNV or USUV IgG antibodies. Specimens with the ratio of ≥ 0.8 to < 1.1 were considered as equivocal, while ratio of <0.8 was interpreted as a negative result. Plasma samples with either positive or equivocal results were defined as ‘reactive’. To exclude TBEV IgG seropositivity, as a result of serological cross-reactivity, all reactive samples were further tested for anti-TBEV IgG antibodies by the Euroimmun’s TBEV IgG ELISA kit (EUROIMMUN Medizinische Labordiagnostika AG, Lübeck, Germany). For confirmation of IgG positivity and evaluation of serological cross-reactivity *in house* indirect immunofluorescence (IFA) and microneutralization assays were used. Anti-WNV/USUV/TBEV IgG IFA was performed as described elsewhere [26].

All anti-WNV or anti-USUV IgG ELISA reactive samples were confirmed by microneutralization assay for WNV and USUV in parallel. Heat inactivation of the specimens before testing was performed at 56°C for 30 minutes. Serial 50% dilutions of 50 μL plasma samples were made and 50 μL of constantly diluted virus solution was added to each well. The fixed dose of WNV or USUV was 100 TCID_50_ (tissue culture infective dose 50%). Plasma–virus mixture was incubated for 90 minutes at 37°C and 5% CO_2_ incubator. 100 μL Vero E6 cell suspension in Dulbecco’s Modified Eagle Medium with 4.5 g/L Glucose and L-Glutamin (Lonza Group AG, Basel, Switzerland) and 5% Fetal Bovine Serum (Merck KGaA, St. Louis, MO, United States) was added to each well. The plates were incubated for 5 days at 37°C and 5% CO_2_. Antibody titre was defined as the inversed value of the highest plasma dilution that resulted in ≥50% reduction of the cytopathic effect of the virus. For quality assurance the following controls were used: positive and negative serum controls, negative cell control and virus titration check (i.e., a back-titration of the test dilution of the virus in each assay). Plasma control of each sample without virus was also tested, to exclude the cytotoxic activity of the tested specimen. A more detailed description of the WNV/USUV microneutralizaton protocol is available at: protocols.io [https://dx.doi.org/10.17504/protocols.io.b4cuqsww].

### Statistical analysis

All statistical analysis was performed using the Microsoft Office Excel of Office 2019 Professional Plus and the Social Science Statistics (https://www.socscistatistics.com/) tool. Pearson’s chi-squared test was used to assess the correlation between the seroprevalence in each stratum. The strength of association between the seroprevalence and the cumulative incidence data in each statistical region was measured by Spearman’s Rho non-parametric test.

Statistical significance was defined at the level of *p<0*.*05*.

ArcGIS® software by Esri (ArcGIS desktop 10.8. version 10.8.0.12790, ESRI: Environmental Systems Research Institute, Redlands, CA.) was used to visualize the geographical distribution of the data.

## Results

### Calculation of seroprevalence across all stratums

Among the 3005 plasma samples evaluated for WNV and USUV specific IgG antibodies, 2609 donors were found to be non-reactive, while 396 samples were reactive for anti-WNV IgG, and 317 specimens for anti-USUV IgG in the initial ELISA tests. 274 donors (38.43%) were reactive in both WNV and USUV IgG ELISA assays revealing the high impact of serological cross-reactivity. All reactive samples were further tested for anti-TBEV IgG antibodies by ELISA. Altogether, 173 donors assumed to be TBEV seropositive. To confirm the WNV or USUV seropositivity, *in house* IFA tests and microneutralization assay for WNV and USUV were also performed.

Among the 396 WNV IgG ELISA reactive specimens, 113 were found to be equivocal. Altogether, 71 of the 113 equivocal samples were positive in TBEV IgG ELISA. USUV and WNV seropositivity was confirmed only in 1 and 3 specimens, respectively. Furthermore, out of the 125 USUV IgG ELISA equivocal plasma samples, TBEV and WNV IgG seropositivity could be confirmed in 49 and 18 cases, respectively.

The total number of WNV seropositive donors was 130 (4.32%), while USUV seropositivity was verified in 5 individuals (0.17%). In one case, WNV IgG seropositive result could not be confirmed by neutralization assay, due to the cytotoxic activity of the plasma.

The mean age of the 130 WNV seropositive blood donors was 41.2 years ranging from 18 to 63. There was no significant difference in the proportion of WNV seropositivity among the three age brackets (*p = 0*.*405*) (Table 1). The mean age of the 5 USUV seropositive blood donors was 35.2 years (ranges from 19 to 61 years). Three out of the 5 USUV seropositive blood donors belonged to the first age bracket (18−34 years), while two of them belonged to the third age group (51−61 years) (Table 1). The proportion of the USUV seropositive donors between the two age groups was 0.31% and 0.22%, respectively. No correlation was found between USUV seropositivity and age (*p = 0*.*199*).

**Table 1:** Distribution of the West Nile and Usutu virus seropositive and non-reactive blood donors by age brackets.

Age groups	Non-reactive blood donors no.	Anti-WNV IgG seropositive donors no.	Anti-USUV IgG seropositive donors no.	Total	Proportion of anti-WNV IgG seropositive donors (%)	Proportion of anti-USUV IgG seropositive donors (%)
**18−34**	929	**44**	**3**	976	**4.51**	**0.31**
**35−50**	1088	**42**	**0**	1130	**3.71**	**N/A**
**51−65**	853	**44**	**2**	899	**4.89**	**0.22**

N/A: not applicable, USUV: Usutu virus, WNV: West Nile virus.

Of the 130 WNV seropositive blood donors 71 were males and 59 were females with mean age of 39.5 and 43.2, respectively. Out of the 5 USUV seropositive donors, 3 were males and 2 were females. Altogether, the number of male and female non-reactive donors was n = 1430 and n = 1440, respectively. By comparing the sex ratio between seropositive and non-reactive donors, no statistically significant difference was found either for WNV (*p = 0*.*289*) or for USUV (*p = 0*.*657*).

### Geographical distribution of WNV and USUV seroprevalence

**Fig 1:**
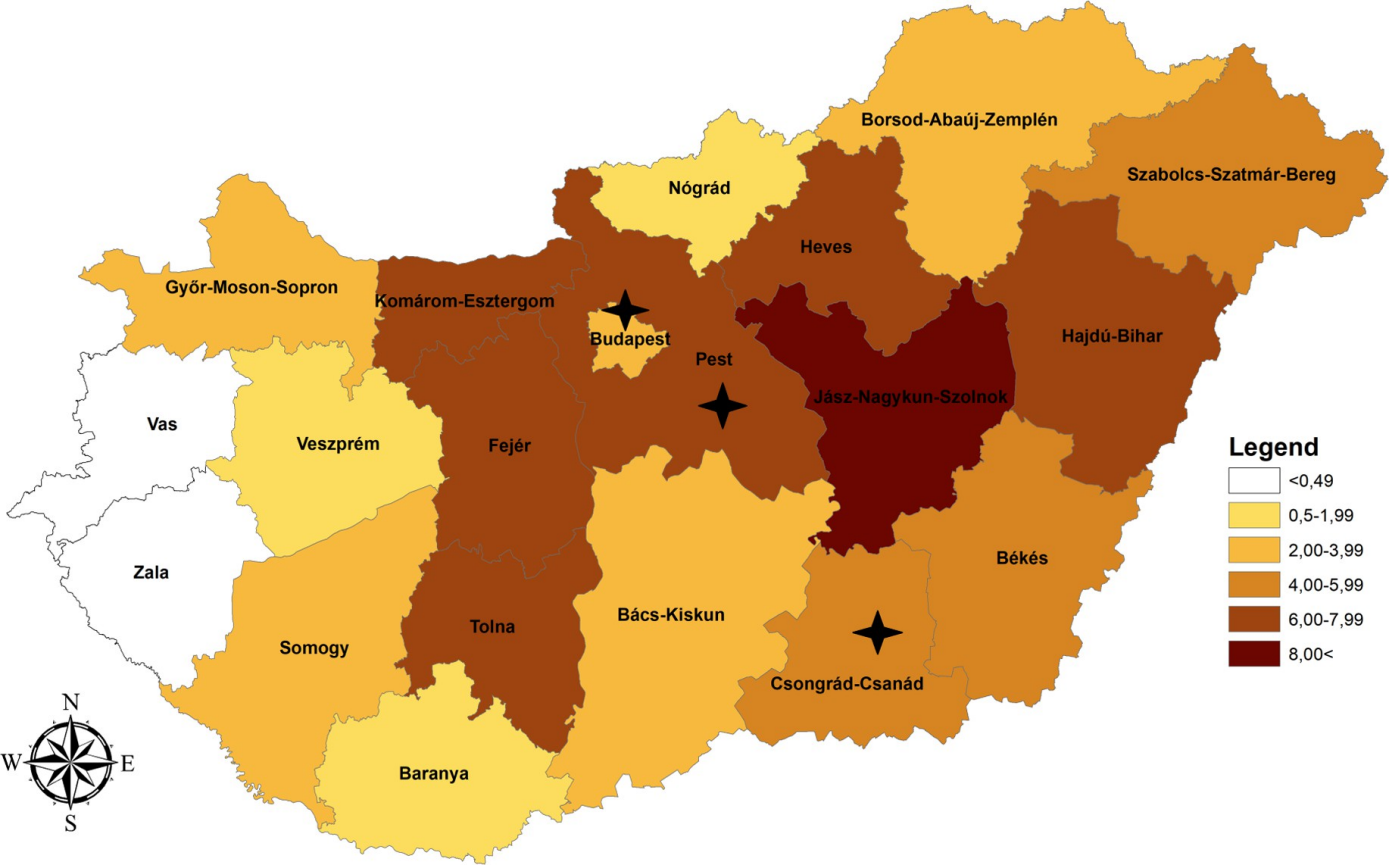
Geographical distribution of the West Nile virus seroprevalence data at NUTS 3 level in Hungary; 2019. NUTS 3 regions where Usutu virus seropositive blood donors (n = 5) were found are indicated by black asterisk.

For the subsequent analysis, all the 3005 samples were categorized by residential areas at NUTS 3 level. The geographical distribution of the 3005 samples were divided into n = 20 NUTS 3 regions. At NUTS 3 level the highest WNV seroprevalence was calculated in Jász-Nagykun-Szolnok (10.71%), followed by Komárom-Esztergom (7.61%), Heves (7.00%), Fejér (6.20%), Hajdú-Bihar (6.17%), Tolna (6.10%) and Pest (6.05%) (Table 2 and Fig 1). Békés (4.95%), Csongrád-Csanád (4.17%) and Szabolcs-Szatmár-Bereg (4.05%) showed also high seroprevalence. Moderate level of anti-WNV IgG prevalence was measured in Bács-Kiskun (3.92%), Budapest (3.73%), Győr-Moson-Sopron (2.84%), Borsod-Abaúj-Zemplén (2.06%) and Somogy (2.00%). In Veszprém (1.92%), Baranya (1.82%) and Nógrád (1.75%) the seropositivity was below the mean value, while in Vas and Zala no WNV seropositive donors were detected (Table 2 and Fig 1).

**Table 2:** West Nile and Usutu virus seroprevalence data in Hungary at NUTS 3 level, in 2019.

No.	Counties (NUTS 3)	Sample no.	Anti-WNV IgG positive no.	Anti-WNV IgG prevalence (%) with 95% CI	Anti-USUV IgG positive no.	Anti-USUV IgG Prevalence (%) with 95% CI
**1**	**Budapest**	536	20	**3.73 (2.28−5.76)**	3	**0.56 (0.12−1.63)**
**2**	**Pest**	379	23	**6.05 (3.84−9.08)**	1	**0.26 (0.00−1.46)**
**3**	**Fejér**	129	8	**6.20 (2.66−12.24)**	0	0.00 (0.00−0.00)
**4**	**Komárom-Esztergom**	92	7	**7.61 (3.03−15.71)**	0	0.00 (0.00−0.00)
**5**	**Veszprém**	104	2	**1.92 (0.19−7.01)**	0	0.00 (0.00−0.00)
**6**	**Győr-Moson-Sopron**	141	4	**2.84 (0.75−7.29)**	0	0.00 (0.00−0.00)
**7**	**Vas**	78	0	0.00 (0.00−0.00)	0	0.00 (0.00−0.00)
**8**	**Zala**	82	0	0.00 (0.00−0.00)	0	0.00 (0.00−0.00)
**9**	**Baranya**	110	2	**1.82 (0.18−6.63)**	0	0.00 (0.00−0.00)
**10**	**Somogy**	100	2	**2.00 (0.19−7.29)**	0	0.00 (0.00−0.00)
**11**	**Tolna**	82	5	**6.10 (1.94−14.27)**	0	0.00 (0.00−0.00)
**12**	**Borsod-Abaúj-Zemplén**	194	4	**2.06 (0.54−5.30)**	0	0.00 (0.00−0.00)
**13**	**Heves**	100	7	**7.00 (2.79−14.45)**	0	0.00 (0.00−0.00)
**14**	**Nógrád**	57	1	**1.75 (0.00−9.94)**	0	0.00 (0.00−0.00)
**15**	**Hajdú-Bihar**	162	10	**6.17 (2.95−11.36)**	0	0.00 (0.00−0.00)
**16**	**Jász-Nagykun-Szolnok**	112	12	**10.71 (5.52−18.73)**	0	0.00 (0.00−0.00)
**17**	**Szabolcs-Szatmár-Bereg**	173	7	**4.05 (1.61−8.35)**	0	0.00 (0.00−0.00)
**18**	**Bács-Kiskun**	153	6	**3.92 (1.42−8.56)**	0	0.00 (0.00−0.00)
**19**	**Békés**	101	5	**4.95 (1.57−11.59)**	0	0.00 (0.00−0.00)
**20**	**Csongrád-Csanád**	120	5	**4.17 (1.32−9.75)**	1	**0.83 (0.00−4.56)**
**TOTAL**		**3005**	**130**	**4.32 (3.61−5.13)**	**5**	**0.17 (0.05−0.39)**

CI: Confidence interval, IgG: Immunoglobulin G, NUTS: Nomenclature of Territorial Units for Statistics, USUV: Usutu virus, WNV: West Nile virus.

USUV seropositive blood donors were found only in two Central Hungarian NUTS 3 regions (Budapest: n = 3; Pest: n = 1) and in a Southern-Hungarian County: Csongrád-Csanád (n = 1) (Table 2 and Fig 1).

### Comparison of the distribution of WNV seroprevalence and the cumulative incidence of WNV cases between 2004 and 2019

**Fig 2:**
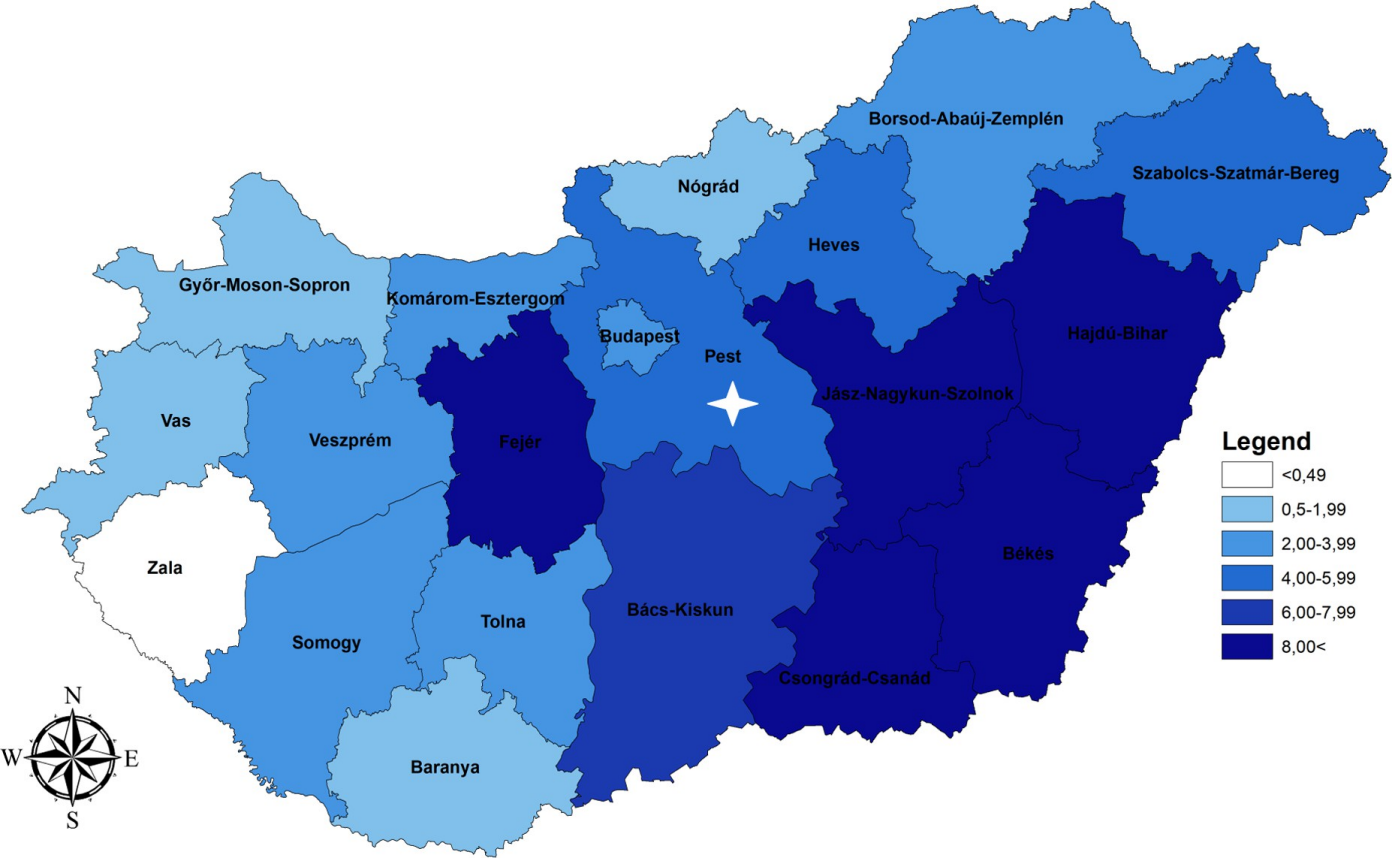
Geographical distribution of the West Nile virus cumulative incidence data at NUTS 3 level in Hungary between 2004 and 2019. NUTS 3 region where acute human Usutu virus infection was detected in 2018 is indicated by white asterisk.

Between 2004 and 2019, altogether n = 459 human WNF or WNND cases were laboratory diagnosed by the National Reference Laboratory for Viral Zoonoses of the NPHC. Overall, the cumulative incidence (WNV cases per 100,000 inhabitants) was 4.62 in the average population (Table 3). Considering the geographical distribution of the incidence rate at NUTS 3 level, Hajdú-Bihar (11.94) proved to be the most affected county, followed by Békés (10.73), Csongrád-Csanád (9.17), Jász-Nagykun-Szolnok (8.72) and Fejér (8.03). In Bács-Kiskun (6.30), Szabolcs-Szatmár-Bereg (5.47), Heves (4.85) and Pest (4.79) the cumulative incidence per 100,000 population was also above the mean value (Table 3 and Fig 2). The capital, Budapest; and five additional counties (Komárom-Esztergom, Veszprém, Somogy, Tolna and Borsod-Abaúj-Zemplén) can be characterized by a moderate level of cumulative incidence, while in four Western-Hungarian counties (Győr-Moson-Sopron, Vas, Zala, Baranya) and in Nógrád (Northern Hungary) the cumulative incidence was much below the average, ranging from 0.35 to 1.95 per 100,000 inhabitants. Detailed results are shown in Table 3 and Fig 2.

**Table 3:** West Nile virus cumulative incidence data between 2004 and 2019, at NUTS 3 level in Hungary.

No.	Counties (NUTS 3)	Average population no. between 2004−2019	Cumulative number of clinical cases between 2004−2019	Cumulative incidence (%) with 95% CI
**1**	**Budapest**	1727891	49	**2.84 (2.10−3.75)**
**2**	**Pest**	1211181	58	**4.79 (3.64−6.19)**
**3**	**Fejér**	423381	34	**8.03 (5.56−11.22)**
**4**	**Komárom-Esztergom**	307016	10	**3.26 (1.56−6.00)**
**5**	**Veszprém**	354459	10	**2.82 (1.35−5.19)**
**6**	**Győr-Moson-Sopron**	449474	6	**1.33 (0.48−2.91)**
**7**	**Vas**	258300	5	**1.94 (0.62−4.53)**
**8**	**Zala**	284124	1	**0.35 (0.00−1.99)**
**9**	**Baranya**	383583	4	**1.04 (0.27−2.68)**
**10**	**Somogy**	317548	9	**2.83 (1.29−5.39)**
**11**	**Tolna**	232081	5	**2.15 (0.68−5.04)**
**12**	**Borsod-Abaúj-Zemplén**	688910	14	**2.03 (1.11−3.41)**
**13**	**Heves**	309030	15	**4.85 (2.71−8.01)**
**14**	**Nógrád**	203231	2	**0.98 (0.10−3.59)**
**15**	**Hajdú-Bihar**	519328	62	**11.94 (9.16−15.30)**
**16**	**Jász-Nagykun-Szolnok**	389861	34	**8.72 (6.04−12.19)**
**17**	**Szabolcs-Szatmár-Bereg**	566328	31	**5.47 (3.72−7.77)**
**18**	**Bács-Kiskun**	523425	33	**6.30 (4.34−8.85)**
**19**	**Békés**	363547	39	**10.73 (7.63−14.66)**
**20**	**Csongrád-Csanád**	414444	38	**9.17 (6.49−12.58)**
**TOTAL**		**9927142**	**459**	**4.62 (4.21−5.07)**

CI: Confidence interval, NUTS: Nomenclature of Territorial Units for Statistics, WNV: West Nile virus.

According to the Spearman’s rank correlation coefficient by normal standards, the association between the WNV seroprevalence and the cumulative incidence in the period of 2004 and 2019 calculated for every NUTS 3 region would be considered statistically significant [*r*_*s*_ *= 0*.*74464*, *p(2-tailed) = 0*.*00017*]. Based on single NUTS 3 data, in five out of the 20 NUTS 3 regions (Győr-Moson-Sopron, Heves, Komárom-Esztergom, Pest, and Tolna) WNV seroprevalence was found to be higher than the cumulative incidence (Tables 2 and 3). The highest difference between the two values was measured in Tolna (Southwest Hungary, Figs 1 and 2), where at 6.10% (95% CI: 1.94−14.27) anti-WNV IgG prevalence, the cumulative incidence was only 2.15 per 100,000 inhabitants (95% CI: 0.68−5.04).

## Discussion

Between 2004 and 2019, cases of WNF and WNND were reported every year in Hungary with a gradually increasing trend in the annual number of diagnosed infections [20]. This tendency was also supported by the results of serological screening of blood donors, as WNV seroprevalence was only 0.61% in 1999/2000, while sixteen years later, it was already 2.19% in the Hungarian population [27]. The highest peak in the number of locally acquired human infections was registered in 2018, when cases have sharply increased in Southern and Central Europe. During the 2018 WNV outbreak, the total number of laboratory confirmed, autochthonous WNF and WNND cases (n = 215) exceeded the cumulative number of cases reported in Hungary between 2004 and 2017 (n = 208). Furthermore, it represents a more than ninefold increase compared with the 2017 transmission period (n = 23). In 2019, however, a much more moderate number of the human WNV infections was reported with the total number of autochthonous cases n = 36. By the end of the 2018 transmission season, the incidence rate (WNV cases per 100 000 inhabitants) was 2.3, which indicated more than a 4.5-fold increase compared with the previous transmission period [20]. The detailed description of the 2018 WNV transmission season in Hungary including the epidemiological data regarding the annual number of reported WNV cases has already been published elsewhere [20]. Our current results show that WNV seroprevalence has been significantly increased in the Hungarian population between 2016 [(2.19% (95% CI: 1.64–2.90)] and 2019 [4.62% (95% CI: 4.21−5.07)] [27]. The study population, however, represented only 0.05% of the total population between 18 and 65 years, which might be a limitation of the screening protocol leading to the underrepresentation of the seropositivity.

Because that WNV mainly causes mild or asymptomatic infections, the number of reported cases may be underrepresented. Doubling the seroprevalence rate in blood samples collected only one year after the largest WNV outbreak in Europe suggests that there has been increased WNV activity in the country.

In Hungary, a passive surveillance system has been operating for the monitoring of avian influenza cases in wild birds since 2006. Under the scope of the avian influenza monitoring programme, bird carcasses have been tested for flaviviruses, like WNV and USUV [22]. Between 2005 and 2015, only sporadic USUV cases and local outbreaks were detected among birds, while an increase in the number of cases could be observed in 2016. In 2015/2016, European lineage 2 of USUV was first detected and since then, it has become prevalent in both neighboring countries, Hungary, and Austria [22]. After the peak in 2016, the number of USUV-positive dead birds has been decreased, according to the surveillance system. All infected birds were found in the Central and Western parts of the country [22]. Although, the sylvatic circulation of USUV was known in Hungary, the first and so far the only one human infection was confirmed in 2018, in the etiology of aseptic meningitis in a patient residing in Central Hungary [20]. So far, there have been no studies performed to evaluate the USUV seroprevalence in the Hungarian human population.

However, there are only limited number of case studies in the literature and a little is known about the long-term sequelae of USUV infection in humans, its geographical distribution may be wider than the distribution of WNV in Europe and several seroprevalence studies provide evidence that human infections might be more common. For example, an unexpected high USUV seroprevalence was found among forestry workers (18.1%) in Northern Italy in 2014−2015 [28], and a further retrospective study determined a higher level of seroprevalence for USUV (6.57%) than for WNV (2.96%) between 2008 and 2011, in Modena, Italy [15], assuming that USUV infection in humans is presumably not a sporadic event.

Despite the low seroprevalence rate (0.17%), confirmation of anti-USUV IgG seropositivity in five blood donors, at three different NUTS 3 regions, reveals that further clinically manifest human USUV cases are expected in Hungary.

WNV seroprevalence in the endemic countries of Europe and the Mediterranean basin has also been investigated in recent studies. In Northern Italy *Pierro et al*. identified 2.08% seroprevalence between 2010 and 2011 [29], while in Greece 2.1% IgG; 0.13% IgM (2012–2013) and in Western Turkey 2.51% IgG seropositivity was measured (2010) [30, 31]. A higher seroprevalence was estimated among blood donors in Qatar where the prevalence of WNV-specific IgG and IgM antibodies were 10.4% and 3.3%, respectively [32].

In Hungary, passive equine surveillance system exists and cases of equine encephalomyelitis due to WNV are notified by the Veterinary Diagnostic Directorate of the National Food Chain Safety Office, which is responsible for the laboratory diagnosis and/or reporting of animal cases of WNV and USUV infections. Both avian infections and outbreaks among equids are regularly reported to the European Animal Disease Notification System (ADNS) of the European Commission (EC). Together with the human infections, seasonal WNV data is integrated within the Surveillance Atlas of Infectious Diseases of the European Centre for Disease Control and Prevention (ECDC) providing an interactive interface to trace the reported autochthonous human and animal cases in Europe and the Mediterranean basin [33]. Although mosquito surveillance is generally considered effective for the prediction of possible animal and human infections, a comprehensive entomological surveillance system is currently not available in Hungary. GMP based regulation ensures the mitigation of WNV risk in blood products in HNBTS. National Public Health and Medical Officer Service notifies all probable WNV cases as well as WNV exposed areas to the HNBTS from the whole country. Moreover, HNBTS follows up seasonal WNV reports issued by ECDC fortnightly and has a direct access to the EU rapid alert system. According to these data, blood donors who permanently live in WNV exposed areas in Hungary or spent at least 24 hours in such territories in Hungary or even in other countries will be deferred for 30 days from blood donation or WNV ID-NAT will be performed from their blood samples. Besides the abundance of the vector and avian populations, the geographical heterogeneity of WNV and USUV epidemiology may also be influenced by the variability of the human population density, as more cases expected in areas with higher densities. On the other hand, the availability of the health care services has also a great impact, as clinical cases that do not fall within the scope of the health care system will not be laboratory investigated or reported. Since the majority of human WNV infections remains asymptomatic and less than 1% develops neurological symptoms with different levels of severity, the laboratory confirmed acute cases represent only the tip of the iceberg. Highest cumulative incidence of WNV was measured mostly in the Eastern és Southern parts of the country, namely in Hajdú-Bihar, Békés, Csongrád-Csanád and Jász-Nagykun-Szolnok counties (Table 2). According to our seroprevalence data, Jász-Nagykun-Szolnok and Hajdú-Bihar are also proved to be highly affected areas (Table 3). For instance, in Hajdú-Bihar there are two clinical centers including a medical university. In regional centers and hospitals having departments specialized for infectious diseases, more attention is being paid for the detection of arbovirus infections. Besides the availability of health care services, certain ecological factors may play a crucial role in the epidemiology of flaviviruses. Both Jász-Nagykun-Szolnok and Hajdú-Bihar counties have many sites providing essential habitat for one or more species of birds, according to the collection of Hungarian bird watching (https://www.hungarianbirdwatching.com/index.html). The highest difference between the two values was calculated in Tolna county (Southwest Hungary) where the cumulative incidence remained much below the WNV seroprevalence. However, this county has a low population density with 50.6–65.0 people per square mile, according to the HCSO’s 2016 data, there is only one hospital which has a unit for infectious diseases. Maybe the availability of health care specialists has a further impact on the relatively low incidence data.

Overall, due to the hidden morbidity and the lack of the nationwide entomological surveillance system, monitoring of the spatial pattern of seroprevalence for both WNV and USUV supports the identification of high-risk areas, raising awareness of health care professionals and the need for preventive measures, such as public health mosquito control and awareness-raising campaigns. Increased surveillance in high-risk areas including the screening of vector, equine and avian populations can further improve the better characterization of the epidemiology of WNV and USUV in the region.

## Supporting information

S1 Data (XLSX)

S2 Data (XLSX)
